# Cefotaxime Loaded Polycaprolactone Based Polymeric Nanoparticles with Antifouling Properties for In-Vitro Drug Release Applications

**DOI:** 10.3390/polym13132180

**Published:** 2021-06-30

**Authors:** Sana Javaid, Nasir M. Ahmad, Azhar Mahmood, Habib Nasir, Mudassir Iqbal, Naveed Ahmad, Sundus Irshad

**Affiliations:** 1 School of Natural Sciences (SNS), National University of Science and Technology (NUST), Islamabad 44000, Pakistan; sana.javaid@sns.nust.edu.pk (S.J.); dr.azhar@sns.nust.edu.pk (A.M.); Habibnasir@sns.nust.edu.pk (H.N.); Mudassir.iqbal@sns.nust.edu.pk (M.I.); 2 Department of Chemistry, University of Wah, Wah Cantt 47040, Pakistan; 3 Polymer Research Lab, School of Chemical and Materials Engineering (SCME), National University of Sciences and Technology (NUST), Islamabad 44000, Pakistan; 4 Department of Pharmacy, Quaid-i-Azam University, Islamabad 44000, Pakistan; natanoli@qau.edu.pk (N.A.); sundusawan02@gmail.com (S.I.)

**Keywords:** cefotaxime, nanoprecipitation, nano-encapsulation, antifouling, nanoparticles

## Abstract

The objective of the present study was to achieve the successful encapsulation of a therapeutic agent to achieve antifouling functionality regarding biomedical applications. Considering nanotechnology, drug-loaded polycaprolactone (PCL)-based nanoparticles were prepared using a nano-precipitation technique by optimizing various process parameters. The resultant nano-formulations were investigated for in vitro drug release and antifouling applications. The prepared particles were characterized in terms of surface morphology and surface properties. Optimized blank and drug-loaded nanoparticles had an average size of 200 nm and 216 nm, respectively, with associated charges of −16.8 mV and −11.2 mV. Studies of the in vitro release of drug were carried out, which showed sustained release at two different pH, 5.5 and 7.4 Antifouling activity was observed against two bacterial strains, Gram-positive *Staphylococcus aureus* and Gram-negative *Escherichia coli*. The zone of inhibition of the optimized polymeric drug-loaded nanoparticle F-25 against both strains were compared with the pure drug. The gradual pH-responsive release of antibiotics from the biodegradable polymeric nanoparticles could significantly increase the efficiency and pharmacokinetics of the drug as compared to the pure drug. The acquired data significantly noted that the resultant nano-encapsulation of antifouling functionality could be a promising candidate for topical drug delivery systems and skin applications.

## 1. Introduction

Microbial infections caused by various pathogenic bacterial strains lead to serious nosocomial infections in patients worldwide [1,2]. Pathogenic bacterial infections due to anomalies in the care and handling of medical equipment and medical textiles, are major sources of mortality and morbidity in developing countries. Used surgical and nonsurgical health care textiles, contaminated with microbial agents, are attributed various nosocomial infections [3,4]. The role of polymeric nanoparticle-associated drug loading and delivery at targeted sites has received a great deal of attention from researchers. Owing to their reduced toxicity, favorable pharmacokinetics, and protection against in vivo and in vitro degradation, the nanoencapsulation of therapeutic agents in biodegradable polymeric nanoparticles, compare to conventional treatments, has provided new routes for research [5,6,7].

Encapsulation of pharmaceutical and therapeutic agents can be achieved through natural and synthetic polymers [8,9]. The hydrophilic nature of antifouling drugs renders them ineffective in treating intracellular infections due to their inability to cross the plasma membrane [10]. Polycaprolactone belongs to a class of polyesters that are promising candidates for the nanoencapsulation of various hydrophilic and hydrophobic drugs [11,12]. The A-chirality of the PCl induces chemical stability in the hydrolysis of long polymeric chains in encapsulation. PCL allows the sustained release of antifouling drug in a controlled manner at a specific site [13].

**Figure 1:**
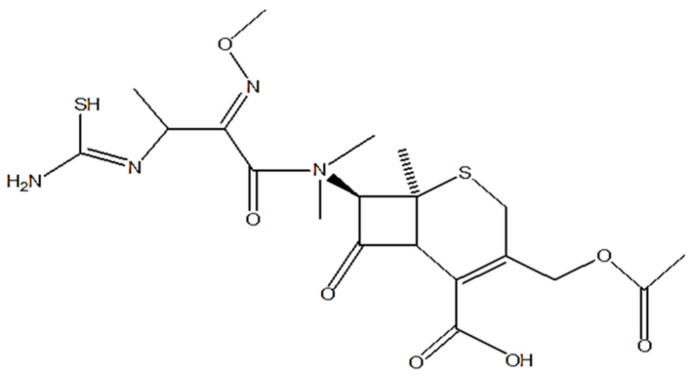
Chemical structure of cefotaxime.

Cefotaxime, a third generation semisynthetic cephalosporin, shown in Figure 1, manifests a broad range antibacterial activity against various bacterial strains [14]. Its bactericidal activity is attributed to its high beta lactamase stability, inhibiting the synthesis of the cell wall. Cefotaxime is effectively used against various microbial strains responsible for skin, lower respiratory, urinary tract, intra-abdominal, bone and joint, and CNS infections [15,16]. Thus, it is considered to be an effective antifouling agent for nanoencapsulation with a high in vitro activity, favorable pharmacokinetics, and a moderate molecular weight [17].

Nanoencapsulation of therapeutic agents is achieved through different mechanisms, including nano-precipitation or the solvent displacement method, emulsification (followed by diffusion/evaporation/coacervation), double-emulsion, polymersome preparation, layer-by-layer and supercritical fluid technology (SCF) [18,19]. A nano-precipitation technique was employed to prepare azithromycin-loaded PLGA nanoparticles for efficient antibacterial activity against *Salmonella typhi* and controlled in vitro release as compared to pure drug [20]. Glycyrrhizin (GL), a hydrophilic drug-loaded chitosan-gum Arabic nanoparticles were prepared to evaluate antibacterial activity as well as in vitro release. Observed analyses showed improved entrapment efficiency and sustained release of glycyrrhizin from the polymeric shell of chitosan-gum Arabic nanoparticles comparative to pure drug [21]. Similarly, polylactide-co-glycolic acid (PLGA)- and polycaprolactone (PCL)-based nanoparticles were prepared for encapsulating etoposide for treating cancer using nanoprecipitation and an emulsification/solvent evaporation technique. Comparing the in vitro release from PLGA co-polymer and PCL nanoparticles individually showed slow and prolonged release up to 48 h, attributed to the hydrophobic nature of PCL NPs. Thus, improved efficacy, safety, and stability of the drug with a good absorption ability was demonstrated [22]. Considering bacterial-resistant isolates, Shabaan et al. synthesized PCL and PLGA NPs for encapsulating imipenem as an antibiotic accompanied by cliastatin to prevent degeneration due to enzymatic effect. Results drawn after subsequent characterization of the nano-formulation clearly showed the effectiveness of PCL-based NPs compared to PLGA NPs in terms of the efficient antibacterial activity of imipenem and in vivo testing further enhanced their therapeutic application [23].

The present work was done with the aim to optimize antifouling formulations for slow and sustained release of antifouling activity. Cefotaxime, a third-generation cephalosporin used for antifouling functionality, was encapsulated through a nanoprecipitation technique. Optimization of the stable formulation in terms of polymer–drug efficacy, homogeneity, and uniformity for prolong periods of time were done using varying concentrations of active ingredients. Particle size and charge were studies using dynamic light scattering technique (DLS). Structural analyses and surface morphology were studied though Fourier transform infrared spectroscopy (FTIR) and scanning electron microscopy (SEM). A UV-Vis spectrometer was used to estimate the encapsulated drug inside the polymeric NPs. The present research work appraised the drug release kinetics with in vitro and slow release of cefotaxime-loaded polycaprolactone-based nanoparticles against two bacterial strains, Gram-positive *S. aureus* and Gram-negative *E. coli*, respectively. Thus, novel developments of cefotaxime-loaded PCL nanoparticles provide an innovative and simple approach for significant antifouling activity in topical and skin-delivery applications. The graphical abstract shows a brief overview of the experimental study and optimization of antifouling formulations using varying parameters accompanied with pH dependent in vitro release and antifouling activity against *E. coli* and *S. aureus*.

## 2. Materials and Methods

### 2.1. Chemicals and Materials

PCL (Polycaprolactone) (Mw-14,000 g/mol), PVA (polyvinyl alcohol) (Mw-3100 g/mol) (Mw/Mn 4.88) and DCM (dichloromethane) (Mw 84.93) of 99.9% purity was purchased from Sigma-Aldrich, Germany. Cefotaxime was obtained from Nectar Life Sciences, India. Ultrapure water (conductivity = 0.055 μS, total dissolved solutes; TDS ~0.00) and deionized water were used for solution preparation and washing for the experimental procedures. Commercial grade materials were used as received. NaCl (Sodium chloride) as buffer saline solution (Sigma Aldrich, Steinheim, Germany) and Mueller-Hinton II Agar (MHA) (Biolab Diagnostics Laboratory, Budapest, Hungary) were used for antibacterial assays. Clinical isolates were acquired from the bacterial strain *Staphylococcus aureus* (ATCC 6538) and *Escherichia coli* (ATCC 8739).

### 2.2. Method

#### 2.2.1. Optimization of Blank Nanoparticles

A nano-precipitation technique was employed to optimize polymeric nano-formulation by developing two phases separately. An organic phase for the blank phase was prepared by dissolving varying concentrations of PCL (25 mg, 50 mg, 75 mg, 100 mg, 150 mg, and 200 mg) in 2 mL of DCM at room temperature to get clear solution. PVA as nonionic surfactants, in varying concentrations (0.1%, 0.3%, 0.5%, 1% and 2%), were induced in 10 mL aqueous phase under constant magnetic stirring at 60 °C for 1 h until a clear solution was obtained. The choice of surfactant and the ratio of organic to aqueous phase, accompanied with other experimental variables, were the key benchmarks for nanoparticles stability.

#### 2.2.2. Optimization of Drug-Loaded Nanoparticles

Drug-loaded nanoparticles were prepared by dissolving 3 mg of cefotaxime in the fixed proportions of PCL and DCM under constant magnetic stirring at 37 °C for approximate 1.5 h to acquire clear solution.

The organic phase was slowly injected into the aqueous phase with constant magnetic agitation using a syringe and a fixed rate of 0.125 mL/min at a constant temperature. The resultant formulation was left in the free space to remove DCM and assess the stability of the nanoparticles according to their physical appearance. Optimization of stable nanoprecipitates was done by varying the concentrations of polymer, surfactant, aqueous/organic phase ratio, temperature, stirring rate, and time, as described in Table 1.

### 2.3. Characterization Techniques

#### 2.3.1. Nanoparticle Size, Charge and Morphology

The optimized formulations were subjected to analyses to determine particle size, charge, and polydispersity index through DLS (dynamic light scattering) (Nano ZS, Malvern Instruments, Worcestershire, UK). Both blank and drug-loaded nano-precipitations were diluted to study the size, charge, and distribution. Each value was taken thrice to acquire a mean value. The size and surface morphology of the nanoparticles were further ascertained via SEM (Model JEOL JSM 6490 LA, Tokyo, Japan). Samples were put on a glass slide and subjected to being coating using gold sputtering, followed by drying, and were placed under the scanning electron microscope stub.

#### 2.3.2. Fourier Transform Infrared Spectroscopy (FTIR)

Fourier transform infrared spectroscopy (FTIR) was used for structural analyses and the identification of functional groups of synthesized formulations. A FTIR spectrophotometer (Shimadzu 8400, Tokyo, Japan) with a wavenumber of 4000 to 400 cm^−1^ was employed for scanning. All formulations were subjected to analyses after making sample pallets using KBr powder and being pressed into a disk.

#### 2.3.3. UV-Visible Spectroscopy

The amount of antifouling drug encapsulated inside the polymeric nanoparticles as well as the liberated amount were estimated using a UV-Vis spectrophotometer (Dynamic, Halo DB- 20, Livingston, UK) at a wavelength of 260 nm. All samples were analyzed by placing them directly into the cuvette inside the spectrophotometer at a specific wavelength. A standard curve was plotted after the drug-released model was fitted, which were used to execute drug loading and release studies.

#### 2.3.4. Study of In Vitro Release Kinetics

The released patterns of drug from the nano-formulation were investigated at pH 5.5 and pH 7.4 for 48 h. A total of 10 mL of formulation was placed in a dialysis bag dispersed in a beaker in 50 mL of phosphate buffer solution (PBS), and maintained under gentle shaking at 37 °C. A total of 2 mL of solution was taken from the beaker after intervals of 0.5, 1, 2, 3, 4, 5, 6, 8, 12, 24, 36, and 48 h, followed by the addition of the same amount of fresh PBS solution for compensation. After subsequent dilutions, samples were investigated under a UV-Vis spectrophotometer at a wavelength of 260 nm to analyze the drug content. Drug release patterns for the nano-formulation were investigated at both pH values. Various kinetic models, such as zero order, first order, Higuchi and Korsmeyer–Papas, were employed to study the in vitro drug release kinetics and ascertain the released mechanisms from a given polymeric system [24].

#### 2.3.5. Antibacterial Assay

Antibacterial activity against two bacterial strains, *Escherichia coli* (ATCC 8739) and *Staphylococcus aureus* (ATCC 6538), were performed using a qualitative agar well diffusion assay. Bacterial cells were first streaked onto freshly prepared nutrient broth and incubated at 37 °C overnight. Bacterial colonies were selected using an inoculated wire loop from the cultured media and put into pre-autoclaved 10-mL saline solution. The bacterial inoculum present in saline solution was vortexed for a uniform distribution and optical densities were adjusted using 0.5 McFarland standards [25]. A total of 25 mL of MHA solution was poured in 9-mm Petri dishes under a streamlined flow hood in the presence of an ethanol lamp. The solidified agar plates were placed into an incubator overnight at 37 °C. A total of 0.1 mL, or 100 microliters, of microbial culture were poured into the center of each Petri dish using a micropipette, followed by swabbing with sterilized cotton buds. Three 6 mm wells constituting one positive control and one negative control, in addition to the sample wells, were formed on the ager plate using sterilized blue tips. Petri dishes were covered with their lids, stacked on top of each other in an upright position and then placed in an incubator at 37 °C. The zone of inhibition around each well was measured and the data were observed as mean ± standard deviation.

## 3. Results and Discussion

### 3.1. Optimization of Synthesized Nano-Precipitation

In the present study, the antifouling formulation was optimized by varying the concentration of constituents (polymer, surfactant, aqueous/organic phase, and drug). Cefotaxime, a third generation cephalosporine, shows a strong affinity in binding with target enzymes, such as those of penicillin-binding proteins, thus inhibiting the synthesis of bacterial cell walls [26]. PCL, a semicrystalline biodegradable polymer, showed slow and sustained release of antifouling activity, which is particularly suitable for topical delivery [22]. PVA, used as a nonionic surfactant, exhibits excellent solubilizing efficiency with PCL-based nano-formulations [27]. Further experimental parameters (stirring speed, stirring time, injection time, and temperature) were studied to achieve an optimized formulation. Thus, a preliminary design to acquire a stable consistency of nano-precipitation was initiated by varying the amounts of surfactant and polymer in fixed proportions of the aqueous and organic phases. After achieving approximate consistency by varying the composition, other process parameters were varied to achieve the desire results. The codes of formulation, variable parameters, ratios of aqueous/organic phase, physical appearance, and status of stability of the formulated nano-precipitations are given in Table 1.

Initially, the amount of PVA as a nonionic emulsifier was varied from 0.1 to 2% in samples F1 to F5, as shown in Table 1. It was found that, by increasing the amount of surfactant and keeping other parameters constant, a uniform consistency of formulation with no globular appearance and precipitation was found, but it does not remain uniform after 24 h, as observed in sample F5. An increased amount of emulsifying agent in the nano-precipitation technique plays a critical role in reducing coalescence of nano-formulation, which was attributed to the reduction in the surface tension between the aqueous and organic phases [28]. Further optimization started with 2% PVA and varying concentrations of polymer, from 50 to 200 mg, for F6 to F10. An increase in the amount of polymer produced a viscous and unstable consistency of nano-precipitation with a settling of particles at the bottom [29]. Magnetic stirring speed was varied from 700 to 1500 rpm in F11 to F19, and non uniformity and instability was observed; thus, sample F12 at a low stirring speed was uniform and more stable with no settling and aggregation of particles in the case of PCL, but there was an increase in the size of the nanoparticles. Nanoparticle size decreased by increasing the rate of stirring, but this affected the rate of solvent evaporation in the nano-precipitation technique and hindered the stability of formulation, causing precipitation and agglomeration, similar to the results for F13 to F19 [30]. Stirring time of the formulation after injection varied from 15 to 20 min for complete evaporation of the solvent. Optimization of the formulation for F21 to F23 was done with the aim to adjust the temperature while injecting the organic phase in an aqueous phase to attain maximum uniformity and stability. In addition to other process parameters, the rate of injecting the organic phase in an aqueous phase is another important factor for the optimization of nano-precipitation, which varied in F24 to F25 from 0.25 to 0.125 mL/min. The slow injection rate of 0.125 mL/min at an optimum temperature of 37 °C in F25 is uniform and highly stable and was selected for loading the drug, which was initiated with a minimum amount of drug, and was then further varied from 3 to 7 mg for F25 to F27. The above results clearly showed that F25, with the least amount of drug loading, was most suitable optimized formulation for the determination of size, surface charge, morphology, antibacterial activity, and drug release kinetics.

### 3.2. Size and Morphology of Nano-Formulation

**Figure 2:**
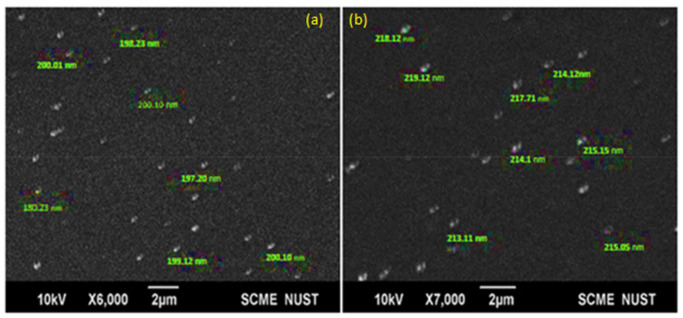
Scanning electron microscopy (SEM) images of (**a**) blank nanoparticles and (**b**) cefotaxime-loaded PCL nanoparticles.

The size of nanoparticles and the morphology of the formulated blank and drug-loaded nano precipitations were characterized through SEM. In Figure 2, SEM images show the spherical morphology of the prepared polymeric nanoparticles with an average size of 200 nm for the blank and 216 nm for the drug-loaded nanoparticles. Uniformity in size and shape of the nanoparticles confirms the absence of coalescence in both blank and drug-loaded nano-precipitations. The marked difference in the size between the blank and drug-loaded polymeric nanoparticles was attributed to the encapsulation of bioactive functional molecules inside the shell of the PCL.

### 3.3. FTIR Analysis

**Figure 3:**
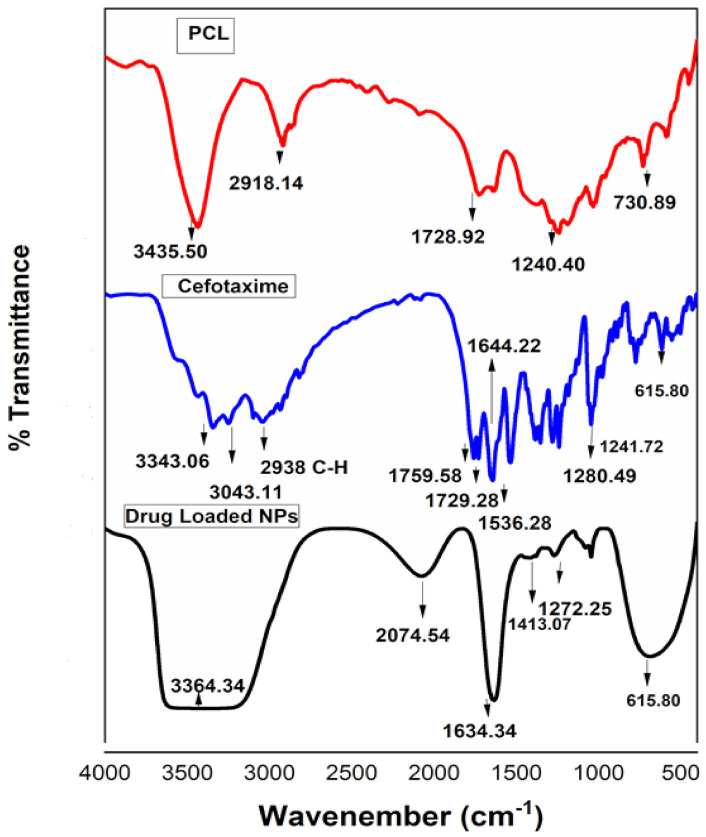
Fourier transform infrared (FTIR) analysis of polycaprolactone, cefotaxime and drug-loaded polymeric NPs.

FTIR analyses of PCL, cefotaxime and antifouling nano-precipitations were observed in the range of 400 to 4000 cm^−1^, as shown in Figure 3. The characteristic peaks of PCL at 3435.50 and 2918.14 cm^−1^ were attributed to O-H and C-H stretching vibrations, while a band appearing at 1728.92 cm^−1^ was attributed to the carbonyl group of ester [31]. The corresponding peaks of NH stretching at 3343.06 and 3043.11 cm^−1^ and C-H stretching at 2938 cm^−1^ appeared in the cefotaxime spectra. C=O stretching of carbonyl (*β*-lactam), carbonyl (carboxylic ester) and carbonyl (amide) were observed at 1759.51, 1729.28 and 1644.22 cm^−1^, respectively [15]. Antifouling emulsion spectra distinctly showed all these peaks. A broad band appeared in the range of 3300 to 3000 cm^−1^ depicting N-H and O-H stretching, and both were present in the PCL and cefotaxime spectra. The other peaks at 1272.25, 1045.36 and 687.36 cm^−1^ corresponded to C-O stretching and C-H bending vibrations, present in the antifouling emulsion spectra. FTIR studies clearly revealed the compatibility between PCL and drug in the nano-precipitation spectra. Such findings confirm chemical stability due to the unaltered structural configurations of functional groups present in PCL and cefotaxime [32,33].

### 3.4. Surface Charge, Particles Size Distribution and Polydispersity Index (PDI)

**Figure 4:**
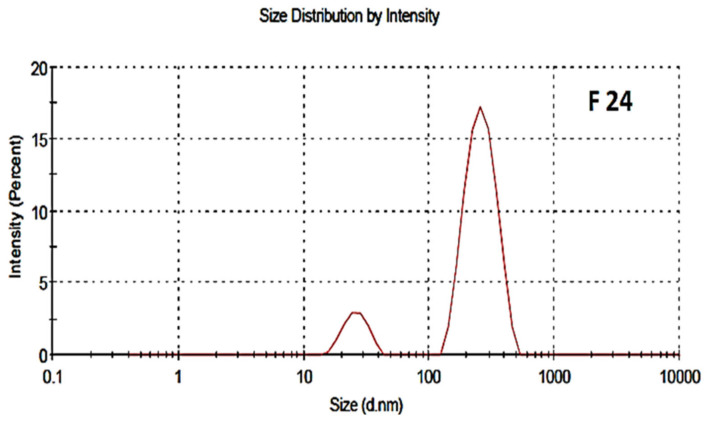
Particle size distribution of blank NPs, F-24.

**Figure 5:**
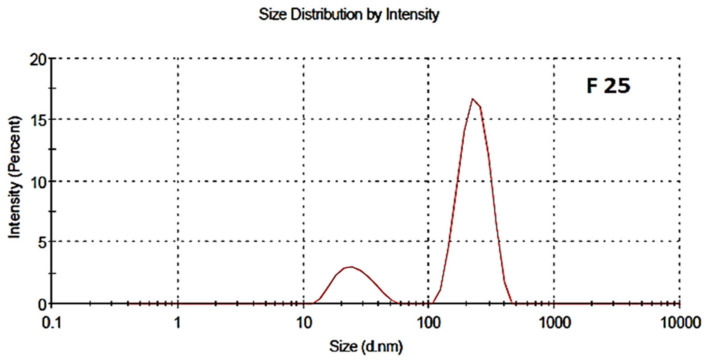
Particle size distribution of drug-Loaded polymeric NPs, F-25.

The particle size distribution and polydispersity index of both the blank and drug-loaded nano formulations are shown in Figure 4 and Figure 5, respectively. The average particle size of the blank and drug-loaded NPs were found to be 200 nm and 216 nm, respectively. The polydispersity index (PDI) was 0.4 and indicated good uniformity and homogeneity in terms of the particle size and distribution of nanoparticles. The PDI lies in the range of 0.1–0.5. Less than 0.1 shows monodispersion while greater than 0.5 shows a high dispersion in terms of particle size.

**Figure 6:**
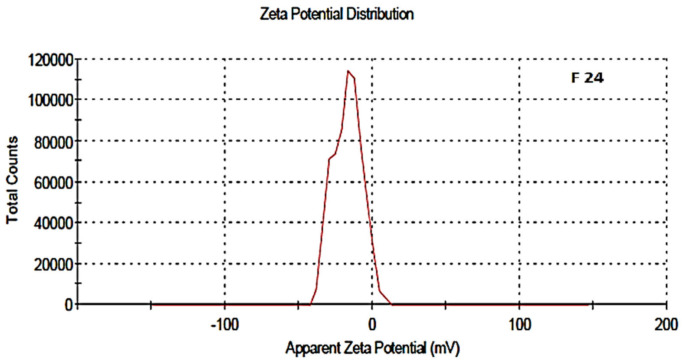
Zeta potential of blank NPs, F-24.

**Figure 7:**
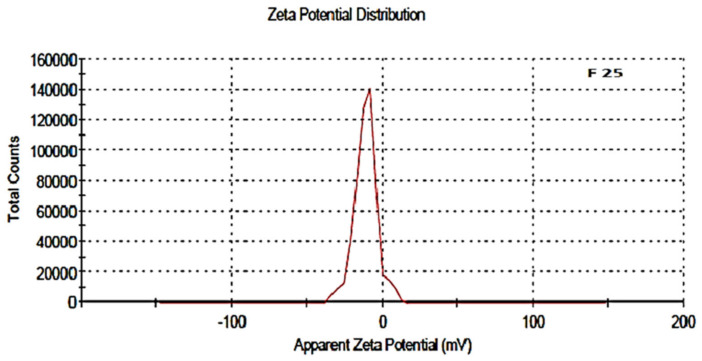
Zeta potential of drug-loaded polymeric NPs, F-25.

The zeta potential was attributed to the stability of nano-formulation. Large positive and negative magnitudes of charge of the nanoparticles described the high stability in the colloidal system, attributed to the large electrostatic repulsion among them. The optimized blank NPs of PCL showed a zeta potential of −16.8 mV, while drug-loaded nanoparticle showed a potential of −11.1 mV, as shown in Figure 6 and Figure 7. Negative zeta potential was due to the presence of the carbonyl group on the surface of the nanoparticles [34,35]. The zeta potential value of the PCL nanoparticles was found to be affected by the method of preparation, i.e., a decreased magnitude of zeta potential, which has already discussed in the case of the nanoprecipitation technique [36]. Owing to the encapsulation of drug in the PCL shell, a decreased value of zeta potential was attributed to the partial adsorption of cefotaxime on the surface and indicated the entrapment of drug inside the PCL nanoparticle.

### 3.5. In Vitro Drug Release Studies

**Figure 8:**
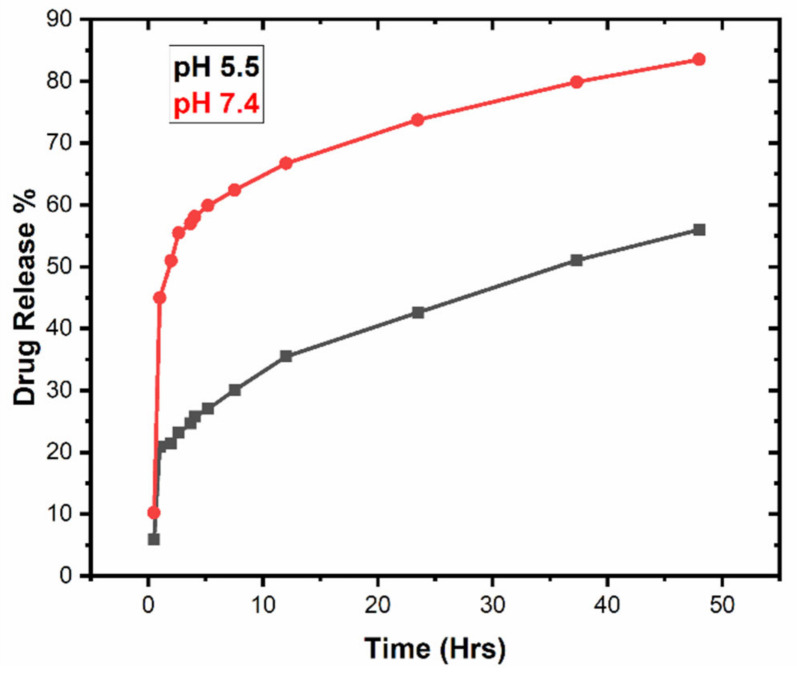
In vitro drug release profile from polymeric nanoparticles at pH 5.5 and pH 7.4.

In vitro drug release studies were performed using the dialysis bag diffusion method for 48 h at two different pH, 5.5 and pH 7.4, in phosphate buffer solution at 37 °C [37]. The percent cumulative drug release versus time plots at pH 5.5 and 7.4 are graphically shown in Figure 8. It is seen from the graph that there was a persistent release of about 56% encapsulated drug from the nano-formulation at pH 5.5, while 83% was released at pH 7.4 within 48 h. A reduced amount of percent cumulative drug, released in acidic medium (pH 5.5), compared to enhanced released in neutral medium (pH 7.4), showed PCL nanoparticles to be promising pH-responsive nano-carriers for hydrophilic drug [38]. A substantial released in the initial 12 h at pH 5.5 (36%) and pH 7.4 (68%) was attributed to rapid dissolution of the adsorbed drug on the surface of the PCL nanoparticles, depending on the surface area to volume ratio [39]. This might be associated with the hydrophilic nature of the drug. On the other hand, a slow and prolonged release in the remaining 36 h was related to the diffusion of drug inside the core of the hydrophobic matrix [40,41].

In vitro release studies of drug from polymeric nanoparticles suggested pH sensitivity in case of PCL nano-carriers. Decreased amounts of percent release at pH 5.5 exhibited a less favorable interaction between drug and dissolution media, as compared to drug–polymer interactions. Thus, the higher release at pH 7.4 reflects the more favorable pharmacokinetics of the drug in the released media [39]. A sustained release of drug from PCL nano-particles was much more desirable for topical application [25]. An unfinished release was observed in case of PCL due to the crystalline and hydrophobic nature, which can be modified by making blends and copolymers using polylactic-co-glycolic acid (PLGA), polylactic acid (PLA) or polyethylene glycol (PEG) [42].

### 3.6. Kinetics of Drug Release

To predict the mechanism of drug release at different pH values, various kinetic models were applied to fit the drug release data of the nano-formulations. Thus, in vitro drug release kinetics was studied by applying the software DD Solver 1.0. Each kinetic model, such as zero order, first order, Higuchi and Korsmeyer–Peppas, describes a different extent of drug pharmacokinetics in the released medium [38]. Zero order kinetic models show that the in vitro release of a drug is independent of the concentration and constant over a certain time, depicted from the zero order kinetic equation, At=A∞+kt. The first order kinetic model$\;(\;\ln\left(\frac{At}{A\infty }\right)=k)$ describes the direct dependence of in vitro release on the concentration of a drug from polymeric nanoparticles. The Higuchi model simplified drug release from the polymeric matrix as the square root of time following the Fickian diffusion method ($\frac{At}{A\infty }=k\surd t$ ). The Korsmeyer–Peppas model is the theorization of the Higuchi model, and is applied where the release mechanism is not fully known from the polymeric system described through the equation,$\;\ln\left(\frac{At}{A\infty }\right)=\ln k+n\;\ln\;\left(t\right)$. Here, the value of n, known as the release or diffusion exponent, depicts the release mechanism of a drug from a polymeric system.

**Figure 9:**
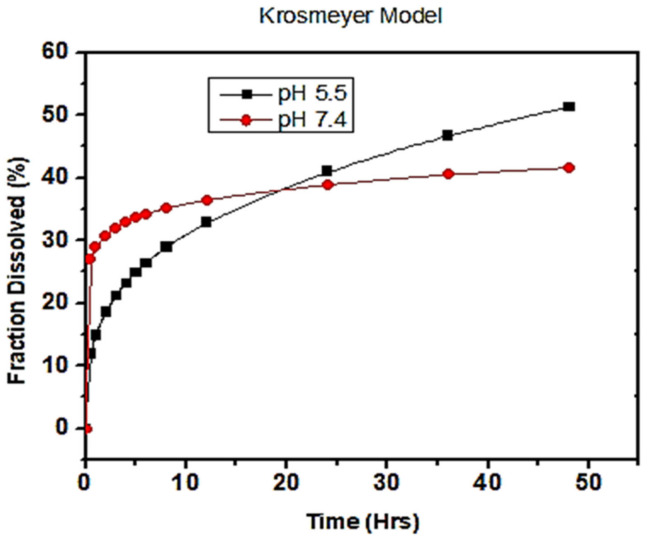
Korsmayer–Peppas kinetic models of drug release from polymeric nanoparticles at pH = 7.4 and pH = 5.5.

The above-mentioned kinetic models were fitted to study the in vitro drug release mechanism of a given polymeric system as a function of R-squared, a coefficient of determination value. The R-squared value gives a goodness of best fit for linear regression models, ranging from 0 to 1. Here, the in vitro release was experimentally studied at pH values of 5.5 and 7.4, and comparatively exhibited favorable interactions between the drug and the release medium. The data analyzed from these kinetic models revealed that the drug release from both pH values (5.5 and 7.4) followed the Korsmeyer–Peppas model with a maximum linearity of R-squared value, as shown in Figure 9. The diffusion exponent or release exponent (n) value from Korsmeyer–Peppas winds up being 0.318 and 0.094 at pH 5.5 and 7.4, given in Table 2, respectively. The n value gives the best fit data for the drug release which is less than 0.5 shows a Fickian diffusion transport mechanism.

### 3.7. In Vitro Antibacterial Assay

**Figure 10:**
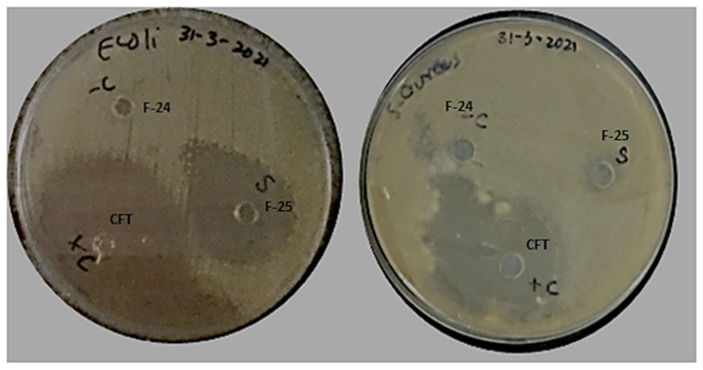
Antibacterial activity of blank NPs (F-24) and drug-loaded NPs (F-25) against *E. coli* and *S. aureus*.

**Figure 11:**
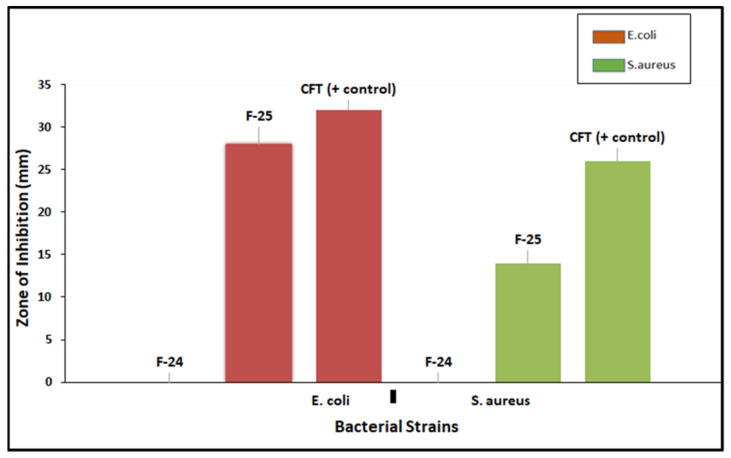
Graphs showing the zone of inhibition (ZOI) of optimized NPs (F-25) against *E. coli* and *S. aureus*.

Antibacterial activity of optimized blank NPs (F-24) and drug-loaded NPs (F-25) against two test strains, *E. coli* and *S. aureus*, were evaluated using an agar well diffusion assay, as shown in Figure 10. The prominent zone of inhibition was shown against both bacterial strains, including pure drug, cefotaxime, as a positive control, and polymeric nano-encapsulated drug (F-25). No zone of inhibition was shown for the polymeric blank nano-precipitation F-24, as a negative control in both plates against *E. coli* and *S. aureus*. Antibacterial activity of F-25 was attributed to the slow and sustained release of drug from NPs from the hydrophobic polymeric shell. The mechanism of bactericidal action was associated with the high beta lactamase stability of cefotaxime which inhibits the growth of the bacterial cell wall [43]. A greater zone of inhibition was shown by F-25 against *E. coli* compared to *S. aureus*, which was significant as seen in the graph in Figure 11. The bactericidal action of F-25 was due to the repulsive electrostatic interaction between the Gram negative *E. coli* and the negatively charged polymeric nanoparticles as compared to the Gram positive *S. aureus* [44]. Further, *E. coli* is more susceptible to antifouling polymeric emulsion due to its thin cell walls compared to those of *S. aureus* [45]. The antibacterial activity of pure drug was higher compared to nano-encapsulated drug NPs (F-25) with a maximum zone of inhibition against both strains. The lesser zone of inhibition of F-25 was attributed to the release kinetics from the nano carriers of drug. Nano encapsulation led to a slow and sustained release of antifouling functionality in 24 h and achieved more favorable pharmacokinetics [46].

## 4. Conclusions

Biodegradable polymeric NPs were synthesized using a nanoprecipitation technique for encapsulating antifouling functional groups for slow release. The surface morphology and particle size were analyzed using scanning electron microscopy and spherical PCL nanoparticles were confirmed. The structural morphology was studied through Fourier transform infrared spectroscopy (FTIR) which validated the characteristic peaks of the polymer and drug in the optimized nano-precipitation. The polydispersity index (PDI) value described the uniformity and homogeneity in terms of particle size distribution. Average particle sizes, charges, and PDI values described a marked difference in the morphology of optimized blank F-24 and drug-loaded F-25 NPs. The zeta potential was found to be negative for PCL nanoparticles, −16.8 mV for blank and −11.1 mV for drug-loaded NPs and exhibited good colloidal stability. In vitro release studies followed the Korsmayer–Peppas model at both physiological pH values of 5.5 and pH 7.4 as a function of R-squared value with a maximum linearity and a high diffusion exponent n value. Cumulative drug release data showed pH-responsive nanocarriers with a maximum release of 83% at neutral pH (7.4) as compared to an acidic pH (5.5) with a 56% release evaluated after 48 h. The slow and sustained release of antifouling drug proves to be the best entity for topical drug delivery application. Antifouling activity were evaluated against Gram positive *S. aureus* and Gram-negative *E. coli* as a function of measured zone of inhibition. It was found that the F-25 nano formulation showed a significant zone of inhibition against *E. coli* and *S. aureus*, with maximum antifouling activity against *E. coli* due to the electrostatic interaction between negatively charged PCL NPs and the thin wall of Gram-negative bacteria. The resultant nano-formulation was proved to be a novel approach for inducing substantial antifouling functionality for cotton textile in the future after evaluating standard antifouling testing and safety protocols.

## Figures and Tables

**Table 1:** Composition of various formulations in the optimization of nanoprecipitation.

Sr No	Aqueous Phase mL	Organic Phase mL	Surfactant %	Polymer mg	Stirring Speed RPM	Stirring Time Min	Temp °C	Injection Rate mL/min	Drug Mg	Observations
F1	10	2	0.1	25	600	10	33	4	0	Non uniform ^a^, unstable ^b^
F2	10	2	0.3	-	-		-	-	-	Non uniform, unstable
F3	10	2	0.5	-	-		-	-	-	Uniform, unstable
F4	10	2	1.5	-	-		-	-	-	Uniform ^c^, unstable
F5	10	2	2	-	-		-	-	-	Uniform, less stable ^d^
F6	10	2	-	50	-		-	-	-	Uniform, unstable
F7	10	2	-	75	-		-	-	-	Non uniform, unstable
F8	10	2	-	100	-		-	-	-	Non uniform, unstable
F9	10	2	-	150	-		-	-	-	Non uniform, unstable
F10	10	2	-	200	-		-	-	-	Non uniform, unstable
F11	10	2	-	25	700		-	-	-	Uniform less stable
F12	10	2	-	-	750		-	-	-	Uniform, more stable ^e^
F13	10	2	-	-	800		-	-	-	Uniform, unstable
F14	10	2	-	-	850		-	-	-	Non uniform, unstable
F15	10	2	-	-	900		-	-	-	Non uniform, unstable
F16	10	2	-	-	950		-	-	-	Non uniform, unstable
F17	10	2	-	-	1000		-	-	-	Non uniform, unstable
F18	10	2	-	-	1250		-	-	-	Non uniform, unstable
F19	10	2	-	-	1500		-	-	-	Non uniform and unstable
F20	10	2	-	-	750	15	-	-	-	Uniform, unstable
F21	10	2	-	-	-	20	35	-	-	Uniform, less stable
F22	10	2	-	-	-	-	37	-	-	Uniform, more stable
F23	10	2	-	-	-	-	40	-	-	Non uniform, unstable
F24	10	2	-	-	-	-	37	8	-	Uniform, more stable
F25	10	2	-	-	-	-	-	16	3	Uniform, highly stable ^f^
F26	10	2	-	-	-	-	-	-	5	Uniform, less stable
F27	10	2	-	-	-	-	-	-	7	Non uniform, unstable

^a^: Precipitates out and globular appearance visible to naked eye; ^b^: formulation remains uniform after 1 h; ^c^: no precipitation and globular appearance visible to naked eye; ^d^: formulation remains uniform up to 24 h; ^e^: formulation remains uniform up to 7 days; ^f^: formulation remains uniform up to 30 days at ambient temperature.

**Table 2:** R-squared and release exponent (n) values acquired from the kinetic study.

pH of Release Medium	Order of Kinetics	R-squared Value R^2^	Release Exponent (n)	Mechanism of Transport
5.5	Korsmayer–Peppas	0.9140	0.318	Fickian diffusion control
7.4		0.9649	0.094	

## Data Availability

All the data will be available to the readers.
